# Mechanistic Modeling of Primaquine Pharmacokinetics, Gametocytocidal Activity, and Mosquito Infectivity

**DOI:** 10.1002/cpt.2512

**Published:** 2022-01-22

**Authors:** Palang Chotsiri, Almahamoudou Mahamar, Richard M. Hoglund, Fanta Koita, Koualy Sanogo, Halimatou Diawara, Alassane Dicko, Julie A. Simpson, Teun Bousema, Nicholas J. White, Joelle M. Brown, Roly Gosling, Ingrid Chen, Joel Tarning

**Affiliations:** ^1^ Mahidol‐Oxford Tropical Medicine Research Unit Faculty of Tropical Medicine Mahidol University Bangkok Thailand; ^2^ Malaria Research and Training Centre Faculty of Pharmacy and Faculty of Medicine and Dentistry University of Science, Techniques and Technologies of Bamako Bamako Mali; ^3^ Centre for Tropical Medicine and Global Health Nuffield Department of Medicine Oxford University Oxford UK; ^4^ Centre for Epidemiology and Biostatistics Melbourne School of Population and Global Health University of Melbourne Melbourne Victoria Australia; ^5^ Radboud Institute of Health Sciences Radboud University Medical Center Nijmegen The Netherlands; ^6^ Department of Epidemiology and Biostatistics University of California San Francisco California USA; ^7^ Global Health Group Malaria Elimination Initiative University of California San Francisco California USA

## Abstract

Clinical studies have shown that adding a single 0.25 mg base/kg dose of primaquine to standard antimalarial regimens rapidly sterilizes *Plasmodium falciparum* gametocytes. However, the mechanism of action and overall impact on malaria transmission is still unknown. Using data from 81 adult Malians with *P*. *falciparum* gametocytemia who received the standard dihydroartemisinin‐piperaquine treatment course and were randomized to receive either a single dose of primaquine between 0.0625 and 0.5 mg base/kg or placebo, we characterized the pharmacokinetic‐pharmacodynamic relationships for transmission blocking activity. Both gametocyte clearance and mosquito infectivity were assessed. A mechanistically linked pharmacokinetic‐pharmacodynamic model adequately described primaquine and carboxy‐primaquine pharmacokinetics, gametocyte dynamics, and mosquito infectivity at different clinical doses of primaquine. Primaquine showed a dose‐dependent gametocytocidal effect that precedes clearance. A single low dose of primaquine (0.25 mg/kg) rapidly prevented *P. falciparum* transmissibility.

**Study Highlights**

**WHAT IS THE CURRENT KNOWLEDGE ON THIS TOPIC?**

☑ The World Health Organization (WHO) suggests adding a single low dose of primaquine to the standard artemisinin combination treatment for malaria transmission blocking effects. Primaquine is highly effective against the mature gametocytes. The gametocyte killing rate is dose‐dependent. Gametocyte sterilization precedes gametocyte clearance. The major adverse effect of primaquine administration is hemolysis in G6PD deficient individuals.

**WHAT QUESTION DID THIS STUDY ADDRESS?**

☑ What are the population pharmacokinetic and pharmacodynamic properties of primaquine? What is the relationship between primaquine and carboxyprimaquine plasma concentrations and transmission blocking effects? How can this mechanistic pharmacokinetic and pharmacodynamic model inform an appropriate dosing strategy?

**WHAT DOES THIS STUDY ADD TO OUR KNOWLEDGE?**

☑ This study is the first population pharmacometric study of primaquine to assess pharmacokinetic properties, gametocyte clearance, and mosquito infectivity. Primaquine and carboxy‐primaquine were modeled simultaneously using a drug‐metabolite model. Different gametocyte reduction rates were found in the different primaquine dosing groups and were modeled as a concentration‐dependent gametocyte killing effect. Mosquito infectivity was proportional to the density of viable mature gametocytes. Higher primaquine doses resulted in rapid gametocyte clearance and rapid reduction in mosquito infectivity.

**HOW MIGHT THIS CHANGE CLINICAL PHARMA‐COLOGY OR TRANSLATIONAL SCIENCE?**

☑ This study provided a mechanistic understanding of primaquine’s pharmacokinetic‐pharmacodynamic properties. Even though increasing primaquine doses resulted in a shortening of the period of malaria transmissibility, the clinical impact of a high dose (0.5 mg/kg) was not substantially greater than that of the lower dose (0.25 mg/kg). The primaquine dose of 0.25 mg/kg is considered as an appropriate dose to block *P. falciparum* transmission.

The 8‐aminoquinoline antimalarial primaquine given over 7–14 days is the recommended antimalarial treatment for radical cure of *Plasmodium vivax* and *Plasmodium ovale* infections. Unlike other antimalarial drugs, primaquine is also highly effective in killing stage V *P. falciparum* gametocytes. A single dose is recommended in addition to standard artemisinin combination treatment (ACT) for the treatment of *P.* *falciparum* malaria in low transmission settings. Primaquine sterilizes gametocytes before they are cleared from the circulation.^1^ The main adverse effect of the 8‐aminoquinoline drugs is dose‐related hemolysis in gluclose‐6‐phosphate dehydrogenase (G6PD) deficient individuals.^1^ The gametocytocidal dose for *P. falciparum* malaria recommended for the past half‐century has been 0.75 mg base/kg (adult dose 45 mg). A recent re‐evaluation of transmission blocking data suggested that one third of this dose (0.25 mg/kg) would be equally effective but with less hemolytic risk.^2^, ^3^

Primaquine phosphate is a 1:1 racemic mixture of (+)‐S‐ and (+)‐R‐enantiomers.^4^ The pharmacokinetic properties of primaquine have been well‐characterized. Primaquine is almost completely absorbed (estimated 96% bioavailability) and rapidly eliminated (terminal half‐life is ~ 5–6 hours).^5^ Several hepatic metabolism pathways have been identified, including via monoamine oxidase (MAO‐A),^6^, ^7^ cytochrome P450 (CYP) isoenzyme (mainly 2D6),^8^ and uridine‐diphosphate‐glucuronic‐acid (UDPGA).^9^, ^10^, ^11^ Carboxy‐primaquine is the major metabolite and it is generated via the MAO‐A mediated pathway. The biologically active metabolites of primaquine formed principally via the CYP2D6 pathway are still poorly characterized. Quinone‐imine and orthoquinone metabolites have been proposed as active metabolites, because they can generate local reactive oxygen species (ROS), notably hydrogen peroxide, through a redox reaction which results in oxidative damage to the parasites (parasiticidal activity) and potentially to the red blood cell (hemolysis).^12^, ^13^ Carboxyprimaquine is generally regarded as biologically inert, although there is some evidence that, like primaquine, it can also form a 5‐6 orthoquinone, and generate reactive intermediates. Co‐administration with commonly used quinoline or structurally related antimalarial drugs can increase primaquine exposures up to 30%.^4^, ^14^

In this article, we quantify the pharmacokinetic properties of primaquine and its metabolite, carboxy‐primaquine using data from 81 Malian patients with *P.* *falciparum* malaria who received the standard dose of dihydroartemisinin‐piperaquine with a single dose of primaquine (a dose between 0.0625 and 0.5 mg/kg) or placebo. A mechanistic pharmacokinetic‐pharmacodynamic model that incorporates gametocyte dynamics was fitted to the peripheral blood gametocyte count data. Gametocyte clearance and mosquito infectivity data associated with different primaquine dosing were simulated to inform a primaquine dosing for blocking human‐to‐mosquito transmission.

## MATERIALS AND METHODS

### Study design and ethical approval

This study was part of a single‐blind, dose‐ranging, adaptive randomized phase II trial of single doses of primaquine combined with dihydroartemisinin‐piperaquine given to adult male patients infected with *P. falciparum* in Mali. All participants were confirmed as gametocytemic by microscopy and had normal G6PD enzyme activity on colorimetric quantification. In the first phase, participants were randomly assigned (1:1:1) to one of the following 3 primaquine doses: 0 mg/kg (placebo; *n* = 16), 0.125 mg base/kg (*n* = 17), and 0.5 mg/kg (*n* = 17). In the second phase, participants were sequentially assigned (1:1) to 0.25 mg/kg (*n* = 15) and 0.0625 mg/kg (*n* = 16) primaquine. All participants also received a standard weight‐based 3‐day treatment regimen of dihydroartemisinin‐piperaquine (40 mg dihydroartemisinin and 320 mg piperaquine per tablet) given as per manufacturer’s guidelines.

Full clinical details and results have been reported elsewhere^15^ and the study inclusion and exclusion criteria can be found in the **Supplementary Methods**. Study approval was obtained from the Ethics Committee of the Faculty of Medicine, Pharmacy, and Dentistry of the University of Science, Techniques and Technologies of Bamako, and the Committee on Human Research at the University of California, San Francisco (UCSF). This study was registered with ClinicalTrials.gov, number NCT01743820.

### Study procedure and blood sampling

After the collection of a baseline sample, the study pharmacist provided primaquine (Sanofi, Laval, Quebec, Canada) by crushing 15 mg primaquine tablets and dissolving in 15 mL drinking water. Primaquine was administered as a nearest dosing regimen assignment. All participants received primaquine with a fatty snack (biscuits) to prevent gastrointestinal side effects. All participants also received the first tablet of a 3‐day course of dihydroartemisinin‐piperaquine (Eurartesim, Sigma‐Tau, Italy). All doses and dosing times were observed and recorded. Participants attended follow‐up appointments at the clinic on days 1, 2, 3, 7, 14, and 28 after the start of treatment.

Gametocyte density measurements were performed on all follow‐up appointments. Blood slides stained with Giemsa were double read by expert microscopists, and the gametocyte numbers in 100 μL of whole blood was estimated using Pfs25 mRNA quantitative real‐time polymerase chain reaction (qRT‐PCR), as described elsewhere.^16^ Microscopy detection and the qRT‐PCR method had a limit of detection of ~ 16 and 3.66 gametocytes/µL, respectively (for details, see **Supplementary Methods**).

Mosquito infectivity was measured on day 0 (pretreatment), and on days 1, 2, and 7, by membrane feeding of *Anopheles gambiae* mosquitoes. Potential mosquito infectivity from person to mosquito was defined as the proportion of dissected mosquitoes with oocysts on day 7 post‐feeding. Participants were classified as infectious if at least one dissected mosquito had at least one oocyst. The mosquito infectivity protocol resulted in a limit of detection of ~ 1%. This mosquito infectivity protocol has been reported previously (for details, see **Supplementary Methods**).^17^

In adult participants, 1.0 mL of whole blood was collected at 0, 1, 2, 3, 4, 6, 8, 12, and 24 hours after the drug administration, and primaquine and carboxy‐primaquine concentrations were measured in plasma using solid‐phase extraction (SPE) followed by liquid chromatography coupled with tandem mass spectrometry. The lower limits of quantification (LLOQs) of the primaquine and carboxy‐primaquine assay were 1.14 mg/mL and 4.88 ng/mL, respectively (for details, see **Supplementary Methods**).

### Population pharmacokinetic‐pharmacodynamic model

Observed primaquine and carboxy‐primaquine concentrations were logarithmically transformed and analyzed simultaneously using nonlinear mixed‐effects modeling using NONMEM version 7.4 (Icon Development Solution, Ellicott City, MD). Pirana version 2.9.0,^18^ Perl‐speaks‐NONMEM version 4.8.0 (PsN),^19^ and R version 3.6.0, were used for automation, model evaluation, and diagnostics during the model‐building process. The first‐order conditional estimation method with interactions (FOCE‐I) was used throughout the population pharmacokinetic analysis. Individual body weight was introduced into the pharmacokinetic model as a fixed allometric function, and all other covariates (age, dose, and CYP2D6 activity score) were investigated by a stepwise addition and elimination approach. The distribution of CYP2D6 activity scores in this population has been reported elsewhere.^20^

**Figure 1:**
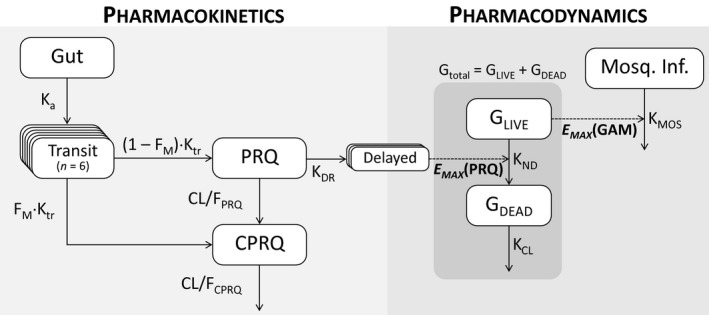
The final pharmacokinetic‐pharmacodynamic model of primaquine (PRQ) and carboxy‐primaquine (CPRQ). The pharmacokinetic model is a drug‐metabolite model with first‐pass metabolism. The pharmacodynamic model consists of a gametocyte model (*G*_LIVE_ and *G*_DEAD_ are live and dead circulating gametocytes, respectively) and a mosquito infectivity model (Mosq. Inf.). The natural death rate of gametocytes (*K*_ND_) is enhanced by exposure to primaquine and modelled by an indirect‐response maximum effect function (E_MAX_(PRQ)). The mosquito infectivity is modeled as a direct‐response maximum effect model (E_MAX_(GAM)). CL/F, total apparent clearance; *F_M_*, fraction of the first‐pass metabolism; *K*_a_, first‐order absorption rate; *K*_CL_, clearance rate of dead gametocytes; *K*_DR_, primaquine delayed effect rate; *K*_MOS_, baseline mosquito infectivity reduction rate; *K*_tr_, transit absorption rate.

Observed gametocyte densities were transformed into their natural logarithms, and nonlinear mixed‐effects modeling was applied to characterize the dynamic relationship between drug concentrations, gametocytemia and mosquito infectivity (**Figure** **1**). Primaquine concentrations (as a surrogate for the bioactive metabolite) were linked to the gametocyte death rate, and implemented as a maximum effect (E_max_) function, resulting in an increased gametocyte killing associated with increasing primaquine dosing. The mosquito infectivity model was combined with the gametocytocidal model, and fitted simultaneously. Mosquito infectivity was described using a one‐compartment model, in which only live gametocytes contributed to mosquito infectivity. The density of live gametocytes was linked to mosquito infectivity by an E_max_ function.

The final pharmacokinetic‐pharmacodynamic model was used to simulate time to negative gametocytemia, and time to negative mosquito infectivity. The final pharmacokinetic‐pharmacodynamic model was used to simulate infectivities associated with placebo, 0.0625, 0.125, 0.25, and 0.5 mg/kg dosing of primaquine.

Further details of the pharmacokinetic‐pharmacodynamic modeling, model diagnostics, and *in silico* simulations can be found in the **Supplementary Methods**.

## RESULTS

This study included 81 patients with *P.* *falciparum* malaria who received dihydroartemisinin‐piperaquine in a clinical trial in combination with different doses of primaquine. The clinical trial has been reported in detail previously.^15^ The baseline clinical and demographic details are shown in **Table** **S1**.

### Population pharmacokinetics of primaquine and carboxy‐primaquine

Primaquine and carboxy‐primaquine plasma concentrations were measured in 10, 3, and 10 patients from the 0.125, 0.25, and 0.5 mg/kg primaquine dose arms, respectively. These plasma concentrations were modeled simultaneously using a drug‐metabolite pharmacokinetic model. A one‐compartment disposition model of primaquine and carboxy‐primaquine proved superior to other disposition models. Primaquine absorption was best described by a transit absorption compartment model with six compartments. Both the first‐order absorption rate (*K_a_*) and the transit absorption rate (*K*_tr_) could be estimated accurately with the rich sampling design covering the absorption phase. Presystemic metabolism was modeled by estimating the fraction of absorbed primaquine converted directly into carboxy‐primaquine (*F_M_*), and the remaining fraction (1–*F_M_*) was introduced in the central compartment as primaquine (**Figure** **1**).^4^ Interindividual variability in pharmacokinetic parameters estimated close to zero were fixed to zero in the final model. Variability in primaquine clearance (CL/*F*_PRQ_) and carboxy‐primaquine volume of distribution (*V*/*F*_CPRQ_) were therefore removed from the final pharmacokinetic model. The correlation between the drug and metabolite residual errors was 0.332 (95% confidence interval (CI): 0.195–0.488). Allometric scaling of the pharmacokinetic parameters improved the model fit to the primaquine and carboxy‐primaquine concentrations (*P* < 0.0001). Adding another primaquine metabolic pathway, (i.e., CYP2D6‐mediated pathway), did not significantly improve the model fit (*P* = 0.2815). Furthermore, including the subjects’ CYP2D6 activity scores as covariates on the clearance of primaquine, implemented either as a covariate on overall clearance or on the alternative primaquine elimination pathway, did not result in a significantly improved fit (objective function value difference (ΔOFV) = −2.03 and −1.16, respectively). No other patient covariates showed significant effects on the pharmacokinetic parameters of primaquine or carboxy‐primaquine.

**Figure 2:**
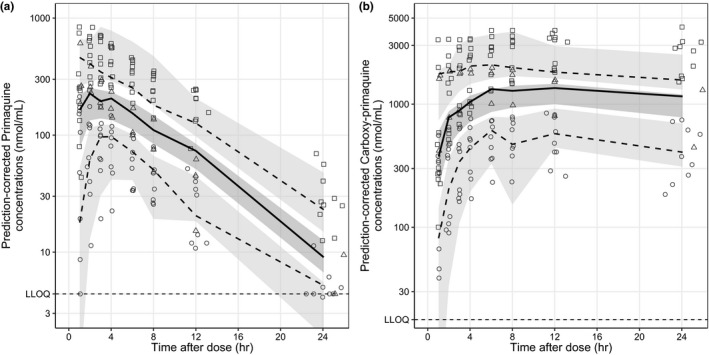
Visual predictive plots of the final population pharmacokinetic model of primaquine (**a**) and carboxy‐primaquine (**b**), stratified by study arms (i.e., circles = 0.125 mg/kg, triangles = 0.25 mg/kg, and squares = 0.50 mg/kg). Solid and dashed lines represent the median, 5^th^ and 95^th^ percentiles of the observations. Shaded areas represent the predictive 95% confidence interval of each percentile. LLOQ, lower limit of quantification.

The final pharmacokinetic model of primaquine and carboxy‐primaquine showed an adequate predictive performance (goodness‐of‐fit plots in **Figure** **S2**, and visual predictive plots in **Figure** **2**). Final pharmacokinetic parameters were estimated with high precision (**Table** **1**).

**Table 1:** Parameter estimates of the final pharmacokinetic‐pharmacodynamic model

Parameters	Population estimates^a^ (%RSE^b^)	95% CI^b^	%CV of IIV^a^ (%RSE^b^)	95% CI^b^
Pharmacokinetic model				
*F*, %	100 (*fixed*)	‐	36.1% (10.3%)	28.5%–43.1%
MTT, hour	0.424 (19.6%)	0.283–0.598	88.8% (10.6%)	72.4%–109%
*K_a_*, hour^−1^	2.27 (26.3%)	1.24–3.56	135% (15.8%)	97.2%–177%
*F_M_*, %	35.9 (9.68%)	29.0–42.8	64.0% (13.0%)	50.9%–82.0%
CL/*F*_PRQ_, L/hour	20.0 (8.58%)	16.9–23.7	‐	‐
*V*/*F*_PRQ_, L	144 (9.14%)	121–172	15.0% (14.3%)	10.4%–19.0%
CL/*F*_CPRQ_, L/hour	1.06 (12.3%)	0.783–1.33	40.7% (14.5%)	31.7%–53.7%
*V*/*F*_CPRQ_, L	35.4 (8.66%)	30.4–42.0	‐	‐
Gametocytocidal model				
*F*_GAM_, %	100 (*fixed*)	‐	27.4% (11.1%)	22.6%–33.3%
*K*_ND_, hour^−1^	0.0022 (10.2%)	0.0018–0.0029	51.9% (13.1%)	37.9%–63.0%
*K*_CL_, hour^−1^	0.0247 (7.78%)	0.0224–0.0308	43.4% (12.6%)	34.1%–57.4%
*K*_DR_, hour^−1^	1.02 (25.3%)	0.677–1.66	‐	‐
E_max_(PRQ), fold	84.0 (27.4%)	61.9–160	89.9% (12.8%)	63.4%–109%
EC_50_(PRQ), nmol/mL	0.0001 (*fixed*)	‐	‐	‐
SLOP, fold change per 1 mg/kg	2.44 (26.5%)	1.05–2.84	‐	‐
Mosquito infectivity model				
*K*_MOS_, hour^−1^	0.0043 (26.0%)	0.0022–0.0067	‐	‐
E_max_(GAM), %	100 (*fixed)*	‐	‐	‐
EC_50_(GAM), gametocytes/μL	1580 (19.3%)	992–2070	181% (9.91%)	143%–213%
Residual error models				
σ_PRQ_	0.0539 (6.55%)	0.0421–0.0708	‐	‐
σ_CPRQ_	0.0046 (7.16%)	0.00357–0.00611	‐	‐
ρ_PRQ~CPRQ_	0.322 (10.86%)	0.196–0.440	‐	‐
σ_prop_	0.287 (3.69%)	0.249–0.335	‐	‐
σ_inf_	0.00249 (4.88%)	0.00221–0.00320	‐	‐

CL/*F*_PRQ_, oral clearance of primaquine; CL/*F*_CPRQ_, oral clearance of carboxy‐primaquine; E_max_(PRQ), maximum primaquine effect on the gametocyte clearance; EC_50_(PRQ), primaquine in the effect compartment at 50% of E_max_(PRQ); E_max_(GAM), maximum gametocyte effect on mosquito infectivity; EC_50_(GAM), gametocyte density associated with 50% of E_MAX_(GAM); *F*, relative bioavailability of primaquine; *F*_GAM_, interindividual variability on the observed gametocytes at enrollment; *F_M_*, fraction of primaquine first‐pass metabolism; *K_a_*, first‐order absorption rate constant; *K*_ND_, gametocyte natural death rate; *K*_CL_, clearance rate of dead gametocytes; *K*_DR_, primaquine delayed rate; *K*_MOS_, baseline mosquito infectivity reduction rate; MTT, mean absorption transit time; *V*/*F*_PRQ_, central volume of distribution of primaquine; *V*/*F*_CPRQ_, central volume of distribution of carboxy‐primaquine; σ_PRQ_, unexplained residual errors of primaquine concentrations; σ_CPRQ_, unexplained residual errors of carboxy‐primaquine concentrations; ρ_PRQ~CPRQ_, correlations of primaquine and carboxy‐primaquine residual errors; SLOP, slope of a linear relationship between dose and E_max_(PRQ); σ_prop_, unexplained residual proportional error of gametocyte predictions; σ_inf_, unexplained residual proportional error of mosquito infectivity predictions.

^a^ Computed population mean parameter estimates from NONMEM were calculated for a typical patient at a body weight of 70 kg. The coefficient of variation (%CV) of the interindividual variability (IIV) was calculated as $\sqrt{\exp\left(\omega^{2}\right)‐1}\times 100$.

^b^ Computed from the sampling important resampling (SIR) procedure^34^, ^35^ of the final pharmacokinetic model with 5 iterations of 1,000, 1,000, 1,000, 2,000, and 2,000 number of samples from the proposal distribution and 200, 200, 400, 500, 500, and 500 resampled parameter vectors.

### Gametocytocidal model

A compartmental nonlinear mixed‐effects model was also developed for the gametocytemia data (**Figure** **1**). Gametocyte development in *P. falciparum* is divided in five stages, where gametocytes in stages I–IV of the development phase are sequestered and then released into the systemic circulation as mature stage V gametocytes. All immature sequestered gametocytes (stages I–IV) were assumed to be eliminated by the antimalarial blood stage therapy (i.e., dihydroartemisinin‐piperaquine), and primaquine alone was assumed to act on the mature stage V gametocytes. Thus, the pharmacodynamic model was developed based on mature gametocyte density measurements only (i.e., the observed circulating parasites), assuming no additional production of stage V gametocytes after initiation of blood stage antimalarial treatment. The gametocyte model consisted of two compartments (alive and dead parasites) and all gametocytes were assumed to be alive at treatment initiation. The gametocytocidal effect of primaquine was implemented as an indirect response model on the transition between alive and dead parasites, resulting in concentration‐dependent killing of gametocytes. Dead gametocytes were modeled as cleared from the circulation independently of primaquine treatment, resulting in a rapid sterilizing effect of primaquine (killing of parasites) and a delayed clearance of circulating gametocytes (removal of dead parasites). The differentiation between gametocyte killing and removal was informed by the mosquito‐infectivity data. Primaquine‐mediated killing of gametocytes was implemented using an E_max_ model. This resulted in a maximum of 84.0‐fold (95% CI: 61.9–160) greater gametocyte death rate in the primaquine arms compared to the natural gametocyte death rate (*K*_ND_) in the placebo arm. Weight‐normalized primaquine dose was a significant covariate on E_max_ (ΔOFV = −8.96), implemented as a linear function, resulting in a faster maximum gametocyte killing at higher primaquine doses. A total of 2.56% and 17.4% of gametocyte density data were below the limit of detection in the placebo arm and primaquine arm, respectively. The higher proportion of data below the limit of quantification in the higher primaquine dosing arm reflected a faster gametocyte clearance in those patients. This mechanistic model was considered the final gametocyte model.

**Figure 3:**
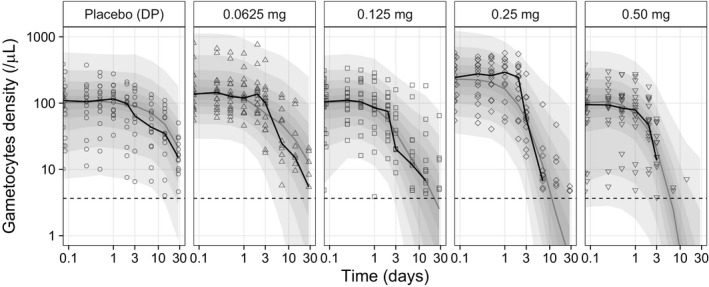
Visual predictive plots of the final population gametocytemia model after primaquine administration, stratified by primaquine dosing group. Grey and black solid lines represent the predicted and observed median gametocyte densities. The outermost shaded areas represent the 95% prediction interval of the final model, with graded degrees of shading for every 10^th^ percentile.

The final model described the parasite and gametocyte density data adequately (**Figure** **S1**, parameters estimated with high precision (% relative standard error < 25%)) with visual predictive plots demonstrating that the model successfully predicts the gametocyte clearance in patients receiving different doses of primaquine or placebo (**Figure** **3**).

### Mosquito infectivity model

**Figure 4:**
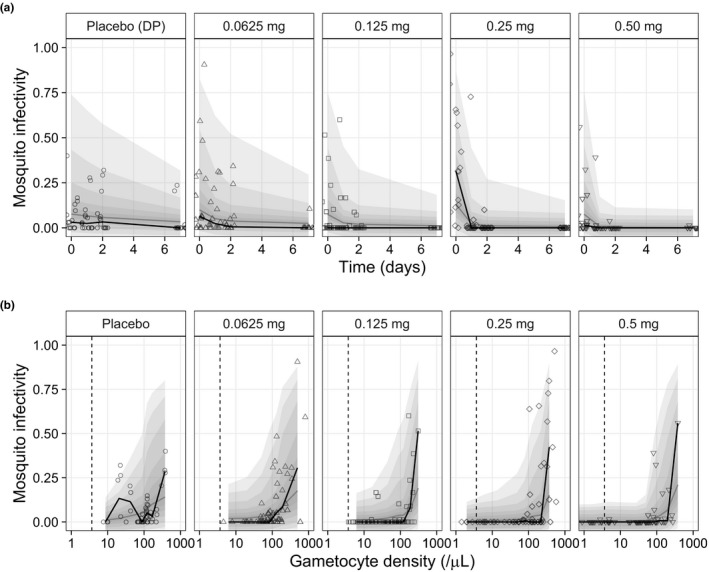
Visual predictive plots of the final mosquito infectivity model after primaquine administration, stratified by primaquine dosing group. (**a**) Mosquito infectivity vs. time after primaquine administration and (**b**) mosquito infectivity vs. gametocytemia. Grey and black solid lines represent the predicted and observed median mosquito infectivity. The outermost shaded areas represent the 95% prediction interval of the final model, with graded degrees of shading for every 10^th^ percentile.

Characterization of the determinants of mosquito infectivity is complex. Enough viable fertile male and female gametocytes must be ingested by the feeding female anopheline mosquito to provide at least one zygote. The infected mosquito must survive long enough for sporozoites to be formed and to populate the salivary glands. The number of mosquitoes with one or more oocysts in their midgut divided by the number of total mosquitoes fed by the patient’s blood at each time point is proportional to the density of viable mature stage V gametocytes in the feeding blood. Approximately 28% of the mosquito experiments could not detect oocysts. This was interpreted as zero mosquito infectivity. Here, the predicted number of live mature gametocytes was linked to mosquito infectivity by an E_max_ model, resulting in a very rapid and immediate decline in infectivity following primaquine treatment. The slower decline in observed gametocytemia resulted from the delayed removal of dead parasites, which are cleared from the circulation independently of primaquine treatment. It was therefore necessary to model mosquito infectivity and gametocyte clearance simultaneously. The E_max_ model assumed 100% mosquito infectivity at high densities of live gametocytes. The estimated gametocyte density associated with 50% mosquito infectivity (EC_50_(GAM)) was 1580 (95% CI: 992–2070) gametocytes/µL in these patients. The parameters in the final mosquito infectivity model were estimated with high precision. Goodness‐of‐fit plot and a visual predictive plot of mosquito infectivity are illustrated in **Figure** **S3** and **Figure** **4**. The final model resulted in a small overprediction in the lowest dose group (**Figure** **4**). However, the parameter estimates described the mosquito infectivity data well with model parameter uncertainties below 10%. The final model predicted the proportion of patients with positive mosquito infectivity precisely (illustrated in **Figure** **S4**).

### Primaquine dosing simulations

**Figure 5:**
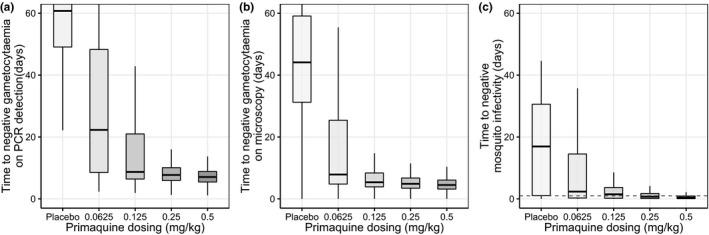
Simulated time to negative gametocytemia by (**a**) PCR detection and (**b**) microscopy detection, and (**c**) time to negative mosquito infectivity. The box‐whisker plots represent the median with inter‐quartile range and the 95% prediction intervals of 500 simulated individuals per dosing group. The dashed line represents the time to negative mosquito infectivity below one day. Assuming the detection limit of qRT‐PCR and microscopy were 3.66 and 16 gametocytes/µL and the detection limit of mosquito infectivity is 1%. qRT‐PCR, quantitative real‐time polymerase chain reaction.

The final developed mechanistic model and its parameter estimates were used to simulate the mosquito infectivity outcomes in a population with different primaquine dosing regimens (*n* = 500 individuals for each dosing regimen). Hypothetical patients with uncomplicated *P*. *falciparum* malaria, presenting with gametocytemia at the start of treatment (geometric mean of 200 gametocytes/μL with a log‐normal distribution with standard deviation of 0.5) were assumed in order to compare the primaquine treatment outcomes after different dosing regimens. The time to negative gametocytemia and the time to negative mosquito infectivity was reduced with increasing primaquine dosage (as illustrated in **Figure** **5**, **Table** **2**). These simulations showed that a single primaquine dose above 0.25 mg/kg can reduce the median time to negative mosquito infectivity below 1 day (median, 0.708 days; 90% CI: 0–13.1 days). An estimated 58.8% and 77.8% of patients from this simulated population receiving 0.25 and 0.5 mg/kg primaquine doses, respectively, had negative mosquito infectivity within one day of treatment. Overall, 90% of patients that received primaquine doses of 0.25 and 0.50 mg/kg were estimated to be noninfectious within 4.19 and 2.00 days, respectively.

**Table 2:** Time to negative gametocytemia and time to negative mosquito infectivity

Primaquine Dosing	Time to negative gametocytemia using PCR (days)	Time to negative gametocytemia using microscopy (days)	Time to negative mosquito infectivity (days)	% Negative mosquito infectivity within 1 day
Placebo (DP)	60.8 (33.3–77.1)	44.1 (10.9–75.6)	16.9 (0–42.6)	25.0%
0.0625 mg/kg	22.3 (5.18–68.7)	7.88 (2.21–60.0)	2.35 (0–35.6)	32.6%
0.125 mg/kg	8.71 (4.63–58.6)	5.38 (2.15–40.4)	1.48 (0–33.0)	40.8%
0.25 mg/kg	7.73 (3.53–34.0)	4.88 (1.37–11.0)	0.708 (0–13.1)	58.8%
0.5 mg/kg	7.08 (3.41–12.3)	4.50 (1.20–8.79)	0.417 (0–3.00)	77.8%

Numbers are presented as median (90% confidence interval). The detection limit of qRT‐PCR and microscopy were assumed to be 3.6 and 16 gametocytes/µL, respectively, and the detection limit of mosquito infectivity was assumed to be 1%.

qRT‐PCR, quantitative real‐time polymerase chain reaction.

## DISCUSSION

This study analyzed pharmacokinetic and pharmacodynamic data from 81 Malian male patients with *P*. *falciparum* malaria and microscopy detected gametocytemia, after receiving placebo or a single dose of primaquine (ranging from 0.0625 to 0.50 mg/kg) in addition to the ACT, dihydroartemisinin‐piperaquine. A combined mechanistic model was developed that describes the pharmacokinetic properties of primaquine and the effects of primaquine on gametocyte dynamics and mosquito infectivity.

Primaquine and carboxy‐primaquine were modeled using a basic drug‐metabolite compartment model, where the population pharmacokinetic model and parameter estimates were similar to those from previous pharmacokinetic reports.^4^ Dense sampling during the absorption phase supported a complex absorption model, consisting of a delayed transit compartment model (6 compartments) with first‐pass metabolism. Primaquine has several metabolism pathways, mediated mainly by CYP2D6 and MAO‐A. Carboxy‐primaquine, the major metabolite (which is biologically inactive), formed via the MAO‐A pathway was described accurately by the drug‐metabolite pharmacokinetic model. The alternative cytochrome P450 (mainly 2D6) pathway generates the bioactive metabolites of primaquine. Low CYP2D6 activity is correlated with primaquine treatment failure.^21^, ^22^ CYP2D6 activity score was evaluated as a covariate on the elimination of primaquine but it was not a significant covariate on primaquine clearance due to a small sample size in the pharmacokinetic arm and lack of information on the primaquine additional metabolism. Additionally, a mechanistic pharmacokinetic well‐stirred model of primaquine^23^ was implemented and evaluated in order to determine the effects of CYP2D6 activity score on the intrinsic clearance of primaquine. However, the intrinsic clearance of primaquine and carboxy‐primaquine through different enzymatic pathways were unidentifiable using the available primaquine and carboxy‐primaquine data in this study.

The exponential decline in gametocyte densities after antimalarial treatment has been described previously by a two‐compartment model, comprising sequestered and circulating gametocytes.^24^, ^25^, ^26^, ^27^ Distriller *et al*. developed a nonlinear mixed‐effects model explaining the dynamic profile of gametocytemia after sulfadoxine‐pyrimethamine administration. A later study showed that the two‐compartment gametocyte model was successful in characterizing the duration of gametocyte carriage after blood‐stage antimalarial treatments.^26^ Administration of an ACT (sulfadoxine‐pyrimethamine plus artesunate or artemether‐lumefantrine) was associated with a four‐fold reduction in gametocyte carriage compared with a non‐ACT antimalarial treatment (sulfadoxine‐pyrimethamine plus amodiaquine; i.e., 55 days, 95% CI: 28.7–107 vs. 13.4 days, 95% CI: 10.2–17.5). Adding a single dose of primaquine (0.75 mg/kg) to the standard ACT exhibited a further four‐fold reduction in the duration of gametocyte carriage (6.3 days (95% CI: 4.7–8.5)). A mechanistic gametocyte model was also developed to describe gametocyte dynamics in human volunteers, infected with *P*. *falciparum* malaria who received piperaquine monotherapy.^27^

In this study, the mechanistic gametocyte model was evaluated with respect to the gametocyte elimination dynamics in patients given different primaquine doses. Gametocytemia was measured using Pfs25 mRNA qRT‐PCR, which detects female gametocytes. As female gametocytes typically make up more than two‐thirds of the total gametocyte biomass,^28^ the observed and predicted gametocyte densities are an underestimate of the total gametocytemia, although comparisons between primaquine dosing groups are unlikely to be biased. Both the observed baseline gametocyte density and mosquito infectivity in the 0.25 mg/kg dose were significantly higher than the other groups (**Figure** **S1**, **Table** **S1**), but this did not affect the gametocyte dynamic model or mosquito infectivity model. The gametocyte dynamic profiles were described using a two‐compartment model to characterize the time‐course of live and dead gametocytes. However, qRT‐PCR cannot distinguish between live and dead gametocytes and the parameters in the structural gametocyte model was therefore not identifiable by fitting measured gametocytemia only. Thus, it was necessary to fit gametocyte and mosquito infectivity data simultaneously, because only live gametocytes can infect a mosquito. A delayed primaquine effect model (indirect‐response model) was used to describe the sustained gametocyte killing effect for several days after drug administration. Although carboxyprimaquine has generally been considered as inert, there is some evidence that a potentially reactive intermediate could be generated via carboxyprimaquine‐5,6‐orthoquinone. In our model, the delayed effect compartment could also be contributed to by carboxyprimaquine as there was a linear correlation between primaquine and carboxyprimaquine exposures. The data generated here does not explain mechanistically why this sustained effect occurs, but it could be a result of reduced viability of gametocytes that were damaged but not killed initially. The observed data, showed a higher gametocyte reduction rate at higher doses of primaquine. However, primaquine concentration‐dependent killing of gametocytes alone was not sufficient to explain these data because all dose groups reached plasma concentrations associated with maximum gametocytocidal effect. Thus, primaquine dosing (i.e., proxy of primaquine exposure) was implemented as a covariate on gametocyte killing. We do not fully understand the mechanism of action of primaquine and the nature and quantities of active metabolite(s), nor is the relationship between gametocyte age and susceptibility well‐characterized.

Male gametocytes may be more sensitive to primaquine than female gametocytes.^29^ The gametocyte‐mosquito infectivity relationship predicted in this study was in agreement with a previous study.^2^ In this study, the relationship between live gametocytes and mosquito infectivity was characterized by an E_max_ function, by fixing 100% mosquito infectivity at high numbers of live gametocytes and estimating the gametocyte density when the mosquito infectivity was reduced to 50% (EC_50_ (GAM)). A combined pharmacodynamic model, describing both gametocyte killing and its relationship to mosquito infectivity, was needed to discriminate between live and dead gametocytes, and accomodate the apparent disconnect between a rapid decline in mosquito infectivity after dosing (reflecting live fertile gametocytes only), but a slower overall gametocyte clearance (live and dead parasites). This simultaneous model of gametocyte density and mosquito infectivity resulted in more stable and robust parameter estimates. A recent pooled meta‐analysis of single low dose primaquine combined with an ACT for the treatment of *P. falciparum* infections suggested that a dose of 0.25 mg/kg is associated with near‐complete transmission‐blocking.^30^ The final pharmacokinetic‐pharmacodynamic model developed here was used to perform *in silico* simulations to evaluate the benefit of increasing primaquine doses, when co‐administered with dihydroartemisinin‐piperaquine (**Figure** **5**, **Table** **2**). These simulations supported the finding that a dose of 0.25 mg/kg is associated with substantially reduced mosquito infectivity (i.e., in a population of gametocytemic patients) with a geometric mean gametocyte density of 100/μL. 58.8% of patients were predicted to be noninfectious within a day of treatment and 90% of patients were predicted to be noninfectious within 4.19 days. Increasing the dose of primaquine to 0.5 mg/kg increased the predicted effect, resulting in 77.8% of patients being noninfectious within 1 day and 90% of the patients being non‐infectious within 2 days. It is important to note that the measure of infectivity in this study was extremely sensitive (≥ 1 oocyst among ≥ 90 blood fed mosquitoes). In field conditions, anopheline mosquito biting rates are usually much lower than this, and daily mosquito survival rates range from 0.68 to 0.98. Thus, in “real world” conditions the impact of single dose primaquine on transmission of malaria from treated individuals at a population level would be expected to be substantially greater than the individual transmissibility impact derived from this modeling exercise.

The potential for iatrogenic hemolysis in G6PD‐deficient patients has limited the use of primaquine. A recent study showed that a single low dose of primaquine (0.25 mg/kg) did not cause harmful hemolytic events in G6PD‐deficient individuals.^31^ Several African countries have adopted the World Health Organization (WHO) policy of co‐administering a single low dose of primaquine (0.25 mg/kg) to their national guidelines for *P*. *falciparum‐*malaria treatment. However, most African countries still do not recommend using primaquine as a transmission blocker in the treatment of *P*. *falciparum* malaria, and most South American countries recommend using a higher primaquine dose (0.75 mg/kg).^32^ Simulations from our mechanistic model showed that a single dose of primaquine at 0.25 mg/kg markedly reduced mosquito infectivity with a relatively minor additional advantage of administering higher doses (0.5 mg/kg). The majority of patients (58.8% after 0.25 mg/kg, and 77.8% after 0.5 mg/kg) were predicted to be noninfectious 1 day after primaquine administration, supporting the WHO recommendation of using primaquine to block malaria transmission.^1^

This study has several limitations. Only primaquine and its abundant metabolite, carboxy‐primaquine, were quantified in this study. The biologically active metabolites (e.g., 5‐hydroxyprimaquine) produced via cytochrome P450 metabolism were not estimated. There is likely to be substantial interindividual variability in the production of these metabolites, which is partly captured by the CYP2D6 genotype. Gametocytemia was measured using Pfs25 mRNA qRT‐PCR, which is specific to the female gametocytes whereas the less abundant male gametocytes are more sensitive to primaquine (and therefore determine transmissibility). Male gametocyte clearance is better correlated with reduction in mosquito infectivity.^2^ The method also does not distinguish the immature noninfectious stage V, mature infectious stage V, or dead gametocytes. Transmission blocking after primaquine administration is determined by gametocyte sterilization, which precedes clearance.^33^ The rate of sterilization was not well‐characterized by the daily sampling schedule and shows substantial interindividual variation. The effect of partner drugs (i.e., dihydroartemisinin‐piperaquine) on the gametocyte clearance could not be determined because all the patients who participated in this study received the same standard dose of dihydroartemisinin‐piperaquine. Dihydroartemisinin has significant gametocytocidal activity, which is not captured in this evaluation. The dynamics of gametocytemia in the oligosymptomatic Malian adults are different to those in more symptomatic patients (i.e., children in areas of high transmission). These oligosymptomatic adults are likely to have a substantially higher proportion of transmissible gametocytes as a result of their chronic untreated malaria infections. In acute symptomatic malaria where immature gametocytes usually predominate, the sterilizing effect should be greater than estimated here for any given gametocyte densities. Mosquito infectivity was defined by the proportion of mosquitos with oocysts in their midgut. Not all oocyst‐positive mosquitoes would develop sporozoites and transmit, especially those with few or single dysmorphic oocysts. This suggests that the inhibition of individual transmissibility was underestimated. Further studies are needed to characterize the relationship among primaquine dose, concentrations of active metabolites, gametocyte sterilization, and the consequent inhibition of oocyst formation and sporozoite production.

## CONCLUSIONS

A mechanistic pharmacokinetic‐pharmacodynamic model successfully described the relationship between primaquine and carboxy‐primaquine pharmacokinetics, gametocyte clearance, and mosquito infectivity. Primaquine rapidly sterilized circulating gametocytes in patients, promptly diminishing the infectiousness of patients’ blood. Higher primaquine doses were associated with a higher gametocyte clearance and lower mosquito infectivity. *In silico* simulations based on data from these presumably chronically gametocytemic adults suggested that ~60% of patients were predicted to be noninfectious within 1 day of administering a single primaquine dose of 0.25 mg/kg. These observations based on stringent assessments of transmissibility in a large number of fed *Anopheles* mosquitoes are not strictly comparable to the treatment of acute symptomatic *P*. *falciparum* malaria in which a much higher proportion of gametocytes are young stage V forms, which are not yet transmissible, but they do allow assessment of relative efficacies and provide strong support for the current low dose recommendation. Adding a single dose of 0.25 mg base/kg of primaquine to the standard ACT treatment for *P. falciparum* infection reduces transmissibility substantially, and should be recommended to accelerate malaria elimination. Further studies are needed to characterize the impact of the relatively small difference in transmission‐blocking activity of primaquine estimated at doses between 0.25 and 0.5 mg/kg.

## FUNDING

This work was supported by the Wellcome Trust (220211); the Bill and Melinda Gates Foundation (INV‐006052); and by a fellowship from the European Research Council (ERC‐2014‐StG 639776) to T.B. The funding bodies did not have any role in the collection, analysis, interpretation of data, writing of the manuscript, or in the decision in submitting the manuscript for publication. For the purpose of open access, the authors have applied a CC BY public copyright license to any Author Accepted Manuscript version arising from this submission.

## CONFLICT OF INTEREST

The authors declared no competing interests for this work.

## AUTHOR CONTRIBUTIONS

All authors wrote the manuscript. A.D., T.B., J.M.B., and R.G. designed the research. P.C., A.M., R.M.H., F.K., K.S., H.D., T.B., I.C., and J.T. performed the research. P.C., R.M.H., J.A.S., T.B., N.J.W., and J.T. analyzed the data.

## Supporting information

Supplementary Material
