# Supplementary material for: Mechanistic Modeling of Primaquine Pharmacokinetics, Gametocytocidal Activity, and Mosquito Infectivity
Source: Clin Pharmacol Ther. 2022 Jan 22;111(3):676–85. doi: 10.1002/cpt.2512 (PMC9302630; doi:10.1002/cpt.2512)
Supplement: Supplementary file 1 — Supplementary Material [file CPT-111-676-s001.pdf]

Supplementary Information

Mechanistic Modelling of Primaquine Pharmacokinetics, Gametocytocidal Activity, and Mosquito Infectivity

Supplementary methods

Study inclusion and exclusion criteria

Inclusion criteria were male sex, age between 5 and 50 years, having at least two *P. falciparum* gametocytes per 500 white blood cells on thick film microscopy (equivalent to ≥ 32 gametocytes per µL, assuming 8000 white blood cells per µL of blood), haemoglobin concentration greater than 80 g/L, and a normal G6PD test result via colourimetric quantification (OSMMR200-D G-6-PG, R&D Diagnostic, Papagos, Greece). The study excluded participants who had taken malaria drugs within 7 days before screening, known allergies to the study drugs, had a serious or chronic illness and diagnosed cardiac arrhythmias. All participants older than 18 years provided written informed consent before screening and enrolment. Parental consent was required for participants younger than 18 years, with children aged 12-17 years also providing assent for inclusion.

Gametocyte density measurements

Gametocyte density measurements were performed on day 0 (pre-treatment), 2 h, 6 h, and 12 h after treatment, and on days 1, 2, 3, 7, 14, and 28. A total of 100 µL of whole blood was collected in L6 buffer (Severn Biotech, Kidderminster, UK), total nucleic acid was extracted using a MagNAPure LC automatic extractor (Total Nucleic Acid Isolation Kit—High Performance; Roche Applied Science, Indianapolis, IN, USA) followed by RQ1 DNaseI digest (Promega, Sunnyvale, CA, USA), cDNA synthesis (High Capacity cDNA Reverse Transcription Kit, Applied Biosystems, Foster City, CA, USA), and molecular quantification of

gametocytes using Pfs25 mRNA quantitative real-time PCR (qRT-PCR), as described elsewhere (1). This qRT-PCR method resulted in a limit of detection of approximately 3.66 gametocyte/µL.

Mosquito infectivity measurements

Mosquito infectivity was measured on day 0 (pre-treatment), and then on days 1, 2, and 7. The mosquito infectivity protocol has been reported previously (2). Venous blood was collected in a heparin tube kept at 37°C and placed in a membrane feeding system within 1 minute. Three cups of 30 *Anopheles gambiae* mosquitos (total 90 mosquitoes, except on day 0 when 180 mosquitoes were used) were fed on the participants blood for 15-20 minutes (2). Blood-fed mosquitoes were then transported to the insectary in Bamako, Mali, and were kept until dissection on day 7 post-feeding for oocyte examination in 1% mercurochrome. Mosquito infectivity from person to mosquito was defined as a proportion of dissected mosquito with oocysts on day 7 post-feeding. Participants were classified as infectious if at least one dissected mosquito had at least one oocyst.

Outcome measures

The primary outcome measure was mosquito infectivity assessed through membrane feeding and measured by oocyst prevalence in mosquitoes dissected on day 7 post feeding. The primary endpoint was compared between the mean of the pre-treatment infectivity and infectivity at 48 hours post-dose. Secondary outcome measures included: the presence of oocysts in mosquitos at other time points, gametocyte prevalence and density determined by microscopy and by molecular methods, primaquine pharmacokinetics, asexual parasite prevalence and density and safety assessment including haemoglobin measurement and evidence of haemolysis.

Drug concentration measurements

In adult participants, 1.0 ml of whole blood was collected in a collection tube containing sodium or lithium heparin as the anticoagulant. The tubes were kept at room temperature (18-25°C) before use. The samples were stored at ambient temperature for up to 2 hours before processing. The blood samples were centrifuged and plasma transferred into screw cap cryovial (Nalgene No.: 5000 0012). Plasma samples were frozen below -20°C, or below -80°C when kept for longer than 2 months. Primaquine and carboxy-primaquine concentrations were measured by the Department of Clinical Pharmacology, Mahidol Oxford University Research Unit, Bangkok. Racemic primaquine and carboxy-primaquine plasma concentrations were measured using solid-phase extraction (SPE) followed by liquid chromatography coupled with tandem mass spectrometry. Quality control samples at low, medium, and high concentrations (i.e. 2.91, 33.7, and 389 ng/mL for primaquine and 14.6, 234, and 3750 ng/mL for carboxy-primaquine) were analysed in duplicate within each batch of study samples to ensure accuracy and precision of the drug assay. Relative standard deviations (%CV) were 7.12%, 6.54%, and 7.89% for primaquine quality control samples, and 5.18%, 4.73%, and 7.93% for carboxy-primaquine quality control samples. The LLOQ of the primaquine and carboxy-primaquine assay were 1.14 ng/mL and 4.88 ng/mL, respectively.

Population pharmacokinetic model

Observed primaquine and carboxy-primaquine concentrations were logarithmically transformed and analysed using non-linear mixed-effects modelling in NONMEM version 7.4 (Icon Development Solution, Ellicott City, MD). Pirana version 2.9.0 (3), Perl-speaks-NONMEM version 4.8.0 (PsN) (4), and R version 3.6.0, were used for automation, model evaluation, and diagnostics during the model building process. The first-order conditional estimation method with interactions (FOCE-I) was used throughout the population pharmacokinetic analysis. Data below the LLOQ were omitted. Population pharmacokinetics

of primaquine and carboxy-primaquine were modelled simultaneously using a drug-metabolite model. Different numbers of primaquine and carboxy-primaquine disposition compartments were evaluated to determine an appropriate drug-metabolite structural model. Different absorption models were evaluated, including the first-order absorption model, transit compartment absorption model, and absorption models with first-pass metabolism. The first-pass metabolism was modelled as a fraction of primaquine converted into and absorbed as carboxy-primaquine (F_M), whereas the remaining fraction ($1 - F_M$) was absorbed unchanged as primaquine. In parallel, a semi-mechanistic pharmacokinetic model (5) using CYP2D6 enzyme activity was also evaluated.

Pharmacokinetic parameters were assumed to be log-normally distributed and therefore on the original scale as follows:

$$\Theta_i = \Theta \times \exp(\eta_{i,\Theta})$$

where Θ_i is individual i^{th} pharmacokinetic parameter estimate, Θ is the typical pharmacokinetic parameter estimate of the population, and $\eta_{i,\Theta}$ is the between-subject variability of parameter Θ of the ith individual. Between-subject variability was assumed to be normally distributed with a zero mean and ω^2 variance. Estimated between-subject variability below 10% or variability estimated with poor precision (%RSE > 50%) were fixed to zero.

A semi-mechanistic pharmacokinetic model which estimated intrinsic primaquine clearance via CYP2D6 and MAO systems (5) was also developed and evaluated. Observed individual CYP2D6 activity scores were modelled as a covariate effect on the primaquine intrinsic clearance. Formation of carboxy-primaquine was hypothesized by the MAO enzymatic activity, then eliminated by the carboxy-primaquine clearance.

Individual body-weight (BW_i) was introduced into the pharmacokinetic model as a fixed allometric function on all volume (exponent of $n = 1.00$) and clearance (exponent of $n = 0.75$) parameters, scaled to the median body weight (61.6 kg) as follows:

$$\Theta_i = \Theta \times \exp(\eta_{i,\Theta}) \times \left(\frac{\mathrm{BW}_i}{61.6}\right)^n$$

All other covariates (age, dose dependent effect, and CYP2D6 activity score) were investigated by a stepwise addition ($p < 0.05$) and elimination ($p < 0.001$) approach. Secondary pharmacokinetic parameter estimates were derived from the *post hoc* pharmacokinetic parameter estimates of the final pharmacokinetic model.

Residual unexplained variability was modelled as an additive error on the log-transformed observed primaquine and carboxy-primaquine concentrations, which is essentially equivalent to a proportional error on an arithmetic scale. Correlation between the drug and metabolite residual unexplained errors was also implemented as the level 2 (L2) data in NONMEM.

Gametocytocidal model

Observed gametocyte densities were transformed into their natural logarithms, and non-linear mixed-effects modelling was applied to characterise the dynamic relationship between drug concentrations, gametocytaemia and mosquito infectivity (***Figure 1***). The structural gametocyte compartmental model was based on the observed mature circulating gametocyte. Artemisinin drugs inhibit young stage-V gametocytes at therapeutic doses, and are highly effective in eliminating immature sequestered gametocytes (6-8). All patients in this trial received a standard 3-day treatment of dihydroartemisinin-piperaquine and the gametocytocidal effects of the artemisinin compound was therefore not considered in the gametocyte density model. The observed gametocyte density was considered a combination of live

and dead gametocytes (G_{LIVE} and G_{DEAD}). The natural death rate (K_{ND}) and gametocyte removal rate (K_{CL}) could be distinguished by using data from both placebo and primaquine treatment arms. Only primaquine was assumed to exhibit a major effect on the mature gametocytes. A transit compartment model (n=3) and an effect compartment were implemented in order to describe the delayed onset and sustained primaquine effect (i.e. hysteresis). The gametocyte density before primaquine dose was implemented as an individual baseline gametocytaemia, and the inter-individual variability of the observed gametocytes (F_{GAM}) was implemented in order to determine the inter-individual variability of the gametocyte reduction profile. Primaquine concentrations (as a surrogate for the bioactive metabolites) in the effect compartment (C_{EFF}) was linked to the parasite death rate, and implemented as an E_{MAX} function, resulting in an increased parasite death rate associated with primaquine treatment. The mg/kg dose of primaquine was introduced as a linear covariate in the gametocyte clearance model to explain the increase in observed maximum parasite clearance at higher doses of primaquine. Differential equations were used to describe the gametocyte dynamics (G_{LIVE} and G_{DEAD}) as follows:

$$\frac{d}{dt}G_{LIVE} = -K_{ND} \cdot \left(1 + \left(\frac{E_{MAX}(\mathrm{PRQ}) \cdot C_{EFF}}{EC_{50}(\mathrm{PRQ}) + C_{EFF}}\right)\right) \cdot G_{LIVE}$$

$$\frac{d}{dt}G_{DEAD} = K_{ND} \cdot \left(1 + \left(\frac{E_{MAX}(\mathrm{PRQ}) \cdot C_{EFF}}{EC_{50}(\mathrm{PRQ}) + C_{EFF}}\right)\right) \cdot G_{LIVE} - K_{CL} \cdot G_{DEAD}$$

where $E_{MAX}(\mathrm{PRQ})$ is the maximum gametocytocidal primaquine effect and $EC_{50}(\mathrm{PRQ})$ is the primaquine concentration associated with 50% of maximum gametocytocidal effect.

Gametocyte density under the limit of detection was modelled using the M6 method, i.e. the first undetected gametocyte measurement in a patient was set to half of the LLOQ value and the remaining measurements were omitted (9). An exponential error model (i.e., modelled as additive error on the log-transformed data) was used to explain the discrepancy between a predicted and observed gametocytaemia measurements.

Mosquito infectivity model

Observed mosquito infectivity was defined as the proportion of dissected mosquitoes containing parasite oocysts. The mosquito infectivity model was combined with the gametocytocidal model (***Figure 1***), and fitted simultaneously. The baseline mosquito infectivity ($Mos.Inf._{BASE}$) was described by a one-compartment model. Only live gametocytes contributed to mosquito infectivity. Therefore, the gametocyte density of live parasites was linked to mosquito infectivity ($Mos.Inf.$) by an E_{MAX} function, as follows:

$$Mos.Inf._{BASE}\ (t) = \exp(-K_{MOS} \cdot t)$$

$$Mos.Inf. = \left(\frac{E_{MAX}(\text{GAM}) \cdot G_{LIVE}(t)}{EC_{50}(\text{GAM}) + G_{LIVE}(t)}\right) \cdot Mos.Inf_{BASE} + \epsilon_{ij}$$

Whereas K_{MOS} is the baseline mosquito infectivity reduction rate, $E_{MAX}(\text{GAM})$ is the maximum mosquito infectivity assumed to be to 100% at very high numbers of live gametocytes, $EC_{50}(\text{GAM})$ is the density of live gametocytes associated with 50% mosquito infectivity, and $G_{LIVE}(t)$ is the predicted density of live gametocytes at time t. An additive constant error (ϵ_{ij}) was used to explain the discrepancy between predicted and observed mosquito infectivity measurements.

Model diagnostics and evaluations

Model fitness was evaluated primarily by the objective function value (OFV; calculated by NONMEM as proportional to -2 × log-likelihood of the data). Model discrimination between two hierarchical models was determined by a likelihood ratio test, based on the Chi-square distribution of the OFV (i.e. p-value < 0.05 when ΔOFV > 3.84, at 1 degree of freedom difference). Potential model misspecification and systematic errors were evaluated by basic goodness-of-fit diagnostics. Eta and epsilon shrinkages were used to assess the ability to detect model misspecifications in goodness-of-fit diagnostics (10). Model robustness and parameter confidence intervals were evaluated by a sampling-important-resampling (SIR) procedure (11, 12). Predictive performances of the final models were illustrated by prediction corrected

visual and numerical predictive checks (n = 2,000) (13). The 5th, 50th, and 95th percentiles of the observed concentrations were overlaid with the 95% confidence intervals of each simulated percentile to detect model bias.

Pharmacokinetic-pharmacodynamic outcome simulations

The final pharmacokinetic-pharmacodynamic model was used to simulate the time to negative gametocytaemia using the quantitative PCR detection and microscopy detection and time to negative mosquito infectivity. The final pharmacokinetic-pharmacodynamic model was also used to simulate different therapeutic outcomes associated with increasing doses of primaquine. Hypothetical patients (n = 500) receiving different primaquine doses (i.e. placebo, 0.0625 mg/kg, 0.125 mg/kg, 0.25 mg/kg, and 0.5 mg/kg) were used to simulate the gametocyte and mosquito infectivity outcomes. These hypothetical patients were assumed to have a population geometric mean baseline gametocytaemia of 200 gametocytes/µL (log-normal distribution; SD = 0.5). It should be noted that this assumes that the hypothetical gametocytaemia had the same properties as that of the asymptomatic adults studied (i.e. in terms of the density/infectivity relationship). We assumed the lower limit of qRT-PCR detection and microscopy to be 3.66 and 16 gametocytes/µL (14), and a mosquito infectivity of 1% was considered to be the detection limit. The time to negative qRT-PCR, microscopy, and mosquito infectivity was simulated and summarised.

Code and data availability

The NONMEM code for the population pharmacokinetic and pharmacodynamic model were provided in the supplementary code below All relevant data are available from the authors upon reasonable request.

NONMEM code for the final PKPD model

```
$PROBLEM    1
$INPUT      ID TIME ODV1 ODV2 ODV3 LNDV1 LNDV2 LNDV3=DV GAME0 ARM CMT
            AMT MDV1 MDV EVID BQL LLOQ INF DISEC ICL IV2 IMTT ICLM IV3
            IFM IF1 IKA IKND IKCL IEMAX IEC50 IGAMM
$DATA       Data.csv
            IGNORE=@
            IGNORE(CMT.EQ.2)   ; Ignore PMQ CMT using predicted individual PK
            IGNORE(CMT.EQ.3)   ; Ignore CPMQ CMT using predicted individual PK

$SUBROUTINE ADVAN13 TOL=9

$MODEL      COMP=(1)  ; GUT compartment
            COMP=(2)  ; Primaquine central compartment
            COMP=(3)  ; Carboxy-primaquine central compartment
            COMP=(4)  ; Absorption transit compartment 1
            COMP=(5)  ; Absorption transit compartment 2
            COMP=(6)  ; Absorption transit compartment 3
            COMP=(7)  ; Absorption transit compartment 4
            COMP=(8)  ; Absorption transit compartment 5
            COMP=(9)  ; Absorption transit compartment 6
            COMP=(10) ; Live Gametocyte compartment
            COMP=(11) ; MOS Infectivity compartment
            COMP=(12) ; Dead Gametocyte compartment
            COMP=(13) ; Delayed effect compartment 1
            COMP=(14) ; Delayed effect compartment 2
            COMP=(15) ; Delayed effect compartment 3

$PK
"FIRST
"COMMON /PRCOMG/ IDUM1,IDUM2,IMAX,IDUM4,IDUM5
"INTEGER IDUM1,IDUM2,IMAX,IDUM4,IDUM5
"IMAX=10000000

  CL    = ICL;
  V2    = IV2;
  MTT   = IMTT;
  CLM   = ICLM;
  V3    = IV3;
  FM    = IFM
  F1    = IF1
  KA    = IKA

  S2    = V2/1000
  S3    = V3/1000

  KTR   = 6/MTT

  K92   = KTR*(1-FM)
  K93   = KTR*FM
  K23   = CL/V2
  K30   = CLM/V3
  K14   = KA
  K45   = KTR
```

```
  K56   = KTR
  K67   = KTR
  K78   = KTR
  K89   = KTR

;;======================================================================
;;                        GAMETOCYTE MODEL
;;======================================================================

  A_0(10) = 0
  A_0(12) = 0

  KND   = THETA(7)*EXP(ETA(7))   ; Gametocyte natural death rate
  KCL   = THETA(8)*EXP(ETA(8))   ; Removal rate of dead gametocyte from the system

  F10   = THETA(9)*EXP(ETA(9))   ; Allow variability of shifting gametocyte curve

  KDR   = THETA(10)*EXP(ETA(10)) ; Rate of the delayed compartment

;;======================================================================
;;                           DRUG EFFECT
;;======================================================================
  DOS = 0
  IF(ARM.EQ.2) DOS = 0.0625
  IF(ARM.EQ.3) DOS = 0.125
  IF(ARM.EQ.4) DOS = 0.25
  IF(ARM.EQ.5) DOS = 0.5

  SLOP   = THETA(11)
  YINT   = THETA(12)

  DOSEF   = SLOP*DOS + YINT

  EMAX    = THETA(1)*EXP(ETA(1))
  EC50    = THETA(2)*EXP(ETA(2))
  GAMM    = THETA(3)*EXP(ETA(3))

;;======================================================================
;;                    MOSQUITO INFECTIVITY
;;======================================================================

  EMAXG   = THETA(4)*EXP(ETA(4))  ; Max infectivity
  ECGA50  = THETA(5)*EXP(ETA(5))

  A_0(11) = 1                     ; Assuming 100% mosquito infectivity at time 0
  K11T0   = THETA(6)*EXP(ETA(6))  ; Reduction rate of mosquito infectivity

$DES
;; Pharmacokinetic model
  DADT(1) = - K14*A(1)
  DADT(2) = K92*A(9) - K23*A(2)
  DADT(3) = K93*A(9) + K23*A(2) - K30*A(3)

  DADT(4) = K14*A(1) - K45*A(4)
  DADT(5) = K45*A(4) - K56*A(5)
```

```
  DADT(6) = K56*A(5) - K67*A(6)
  DADT(7) = K67*A(6) - K78*A(7)
  DADT(8) = K78*A(7) - K89*A(8)
  DADT(9) = K89*A(8) - K92*A(9) - K93*A(9)

  DADT(13) = K23*A(2) - KDR*A(13)
  DADT(14) = KDR*A(13) - KDR*A(14)
  DADT(15) = KDR*A(14) - KDR*A(15)

  ECONC = A(15)/S2
  DEFF = 0
  IF(ECONC.GE.0.0001) THEN
    DELL = 0.0000001
    DFF  = EMAX*((ECONC+DELL)**GAMM)/((EC50+DELL)**GAMM + (ECONC+DELL)**GAMM)
  ENDIF

;; GAMETOCYTE COMPARTMENT
  DADT(10) = - KND*(1+DOSEF*DFF)*A(10)
  DADT(12) = KND*(1+DOSEF*DFF)*A(10) - KCL*A(12)

;; MOSQUITO INFECTIVITY COMPARTMENT
  DADT(11) = -K11T0*A(11)

$ERROR
  PMQCONC  = A(2)/S2
  CPMQCONC = A(3)/S3
  GAM      = A(10) + A(12)
  LGAM     = A(10)
  BMOS     = A(11)

  DEL = 0.0000001

  EFCON = A(15)/S2
  DF   =  EMAX*((EFCON+DEL)**GAMM)/((EC50+DEL)**GAMM + (EFCON+DEL)**GAMM)

  GAMEFF = EMAXG*(LGAM + DEL)/((LGAM + DEL) + (ECGA50 + DEL))
  MOSINF = GAMEFF

  IF(CMT.EQ.10)  IPRED = A(10) + A(12)   ; Predicted gametocyte density
  IF(CMT.EQ.11)  IPRED = A(11)*MOSINF    ; Predicted mosquito infectivity

  IF(CMT.EQ.10.AND.GAM.GT.0) THEN        ; Error model of gametocyte density
    IPRED = LOG(GAM)
    W     = SQRT(SIGMA(1,1))
    Y     = IPRED + EPS(1)
  ENDIF
  IF(CMT.EQ.11) THEN                     ; Error model of mosquito infectivity
    IPRED = A(11)*MOSINF
    W     = SQRT(SIGMA(2,2))
    Y     = IPRED + EPS(2)
  ENDIF

  IRES  = DV-IPRED
  IWRES = IRES/W
```

```
;; Initial estimates
$THETA
(0, 81.3,500)    ; EMAX
(0.0001) FIX     ; EC50
(1) FIX          ; GAMM
(1) FIX          ; EMAXG
(0, 1260,100000) ; ECGA50
(0, 0.0089)      ; K11T0
(0, 0.00237)     ; KND
(0, 0.0268)      ; KCL
(1) FIX          ; F10
(0, 0.902)       ; KDR
(0, 2.37)        ; SLOP
(0) FIX          ; YINT

$OMEGA
 0.76            ; IIV_EMAX
 0 FIX           ; IIV_EC50
 0 FIX           ; IIV_GAMM
 0 FIX           ; IIV_EMAXG
 2.76            ; IIV_EC50_GAM
 0 FIX           ; IIV_K21T0
 0.223           ; IIV_KND
 0.216           ; IIV_KCL
 0.072           ; IIV_F10
 0 FIX           ; IIV_KDR

$SIGMA 0.279     ; Proportional error of gametocyte density
$SIGMA 0.00254   ; Additive error of mosquito infectivity

$ESTIMATION MAXEVAL=9999 PRINT=2 POSTHOC METHOD=1 INTER MSFO=msf1
            NSIG=3 SIGL=9 NOABORT MCETA=500

$COVARIANCE PRINT=E MATRIX=R UNCONDITIONAL

$TABLE ID TIME DV AMT CMT MDV EVID CL V2 KA CLM V3 W ETAS(1:LAST)
       PMQCONC CPMQCONC EFCON GAM IPRED PRED IRES IWRES CL V2 MTT
       CLM V3 FM F1 KA KND EMAX EC50 GAMM CWRES NPDE ARM MOSINF
       GAMEFF DISEC BMOS LLOQ LGAM NOPRINT ONEHEADER FILE=mytab1.tab
```

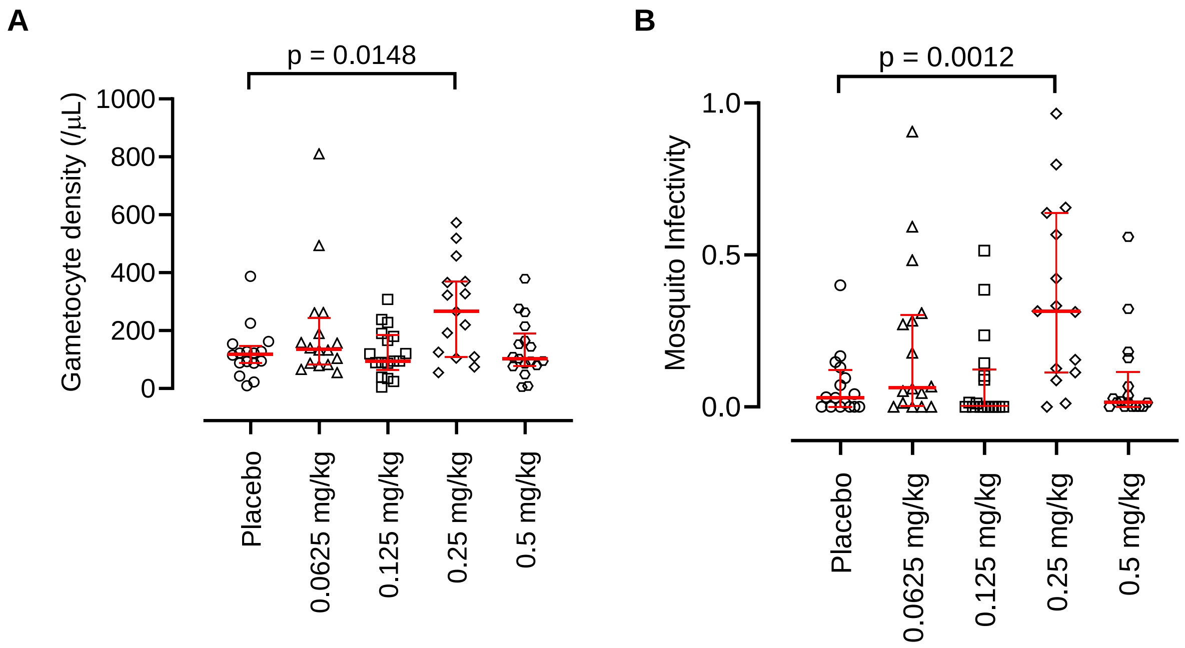

Figure S1. Baseline gametocyte density and mosquito infectivity stratified by the primaquine dosing group. Patients received the 0.25 mg/kg primaquine dose had significantly higher baseline gametocytaemia (p-value = 0.0148, using Mann-Whitney test) and mosquito infectivity (p-value = 0.0012, using Mann-Whitney test) compared to the placebo group. Other dosing group did not exhibit any significant different baseline gametocytaemia and mosquito infectivity to the placebo group.

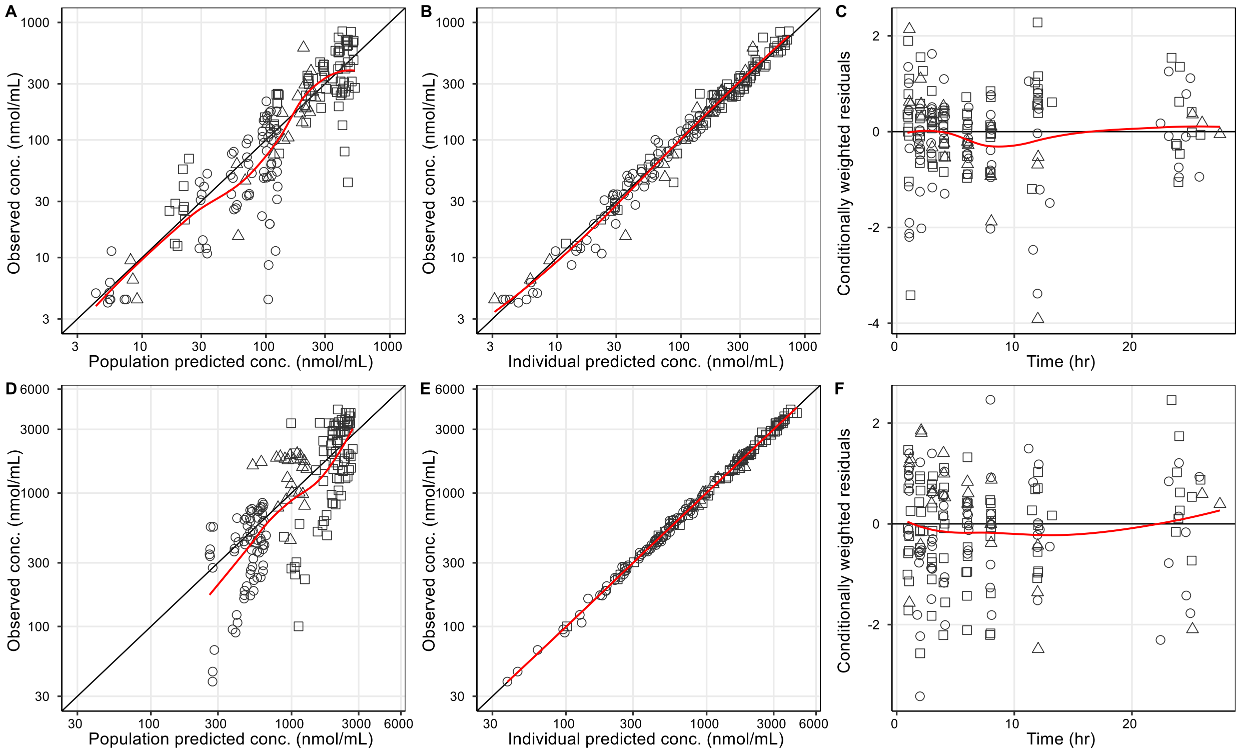

Figure S2. Goodness-of-fit plots of plasma primaquine (A-C) and carboxyprimaquine (D-F) stratified by study arms (i.e. circles = 0.125 mg/kg, triangles = 0.25 mg/kg, and square = 0.50 mg/kg). (A, D) population predictions versus observations, (B, E) individual predictions versus observations, and (C, F) time versus conditionally weighted residuals. Red lines represent locally weighted least-square regressions based on the observed concentrations.

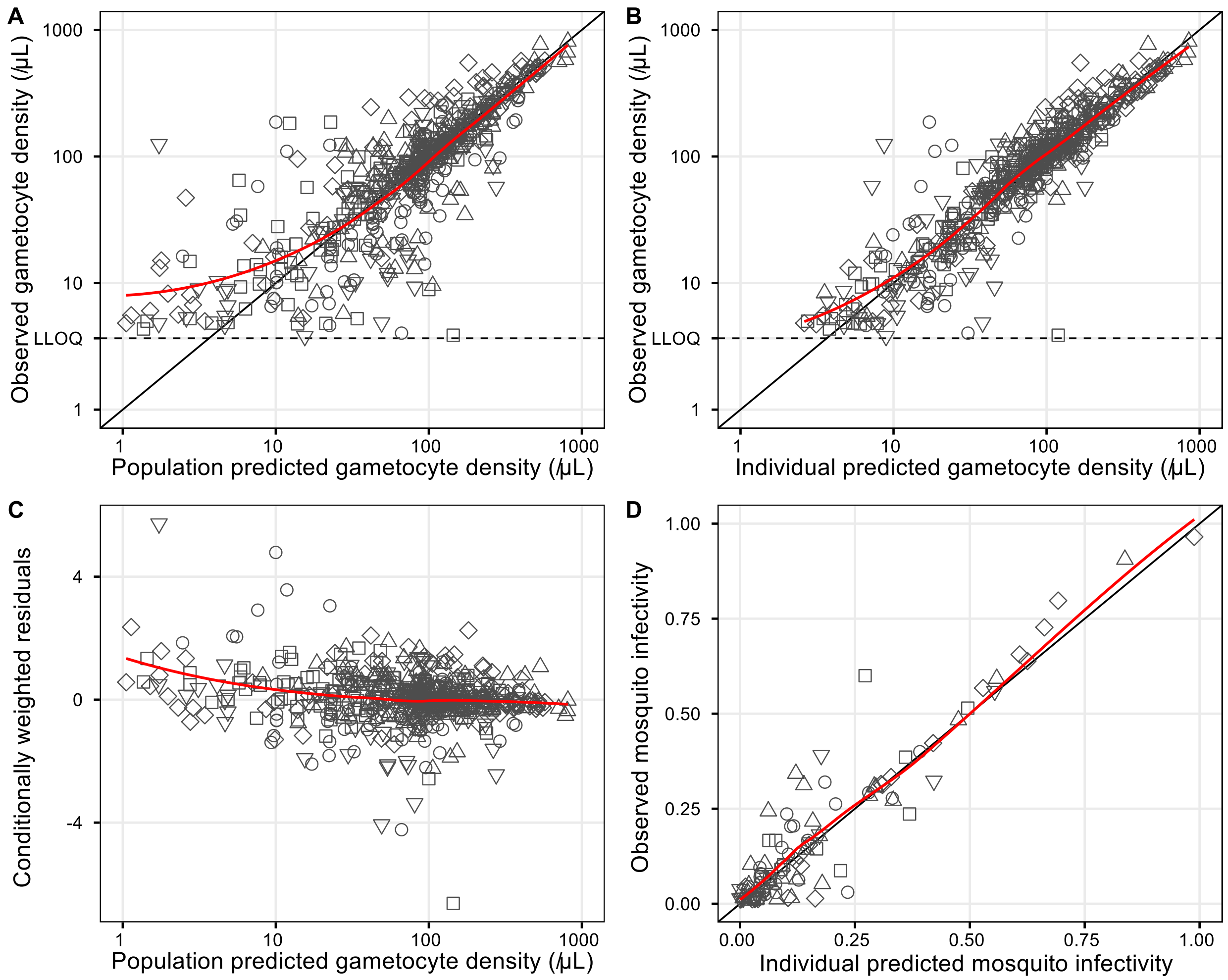

Figure S3. Goodness-of-fit plots of pharmacodynamic model stratified by study arms (i.e. circles = placebo, triangles = 0.0625mg/kg, square = 0.125 mg/kg, diamond = 0.25 mg/kg, and down pointed triangle = 0.50 mg/kg). (A) population predicted gametocyte density versus observations, (B) individual predicted gametocyte density versus observations, and (C) population predicted gametocyte density versus conditionally weighted residuals, and (D) individual predicted mosquito infectivity versus observations. Red lines represent locally weighted least-square regressions based on the observed concentrations.

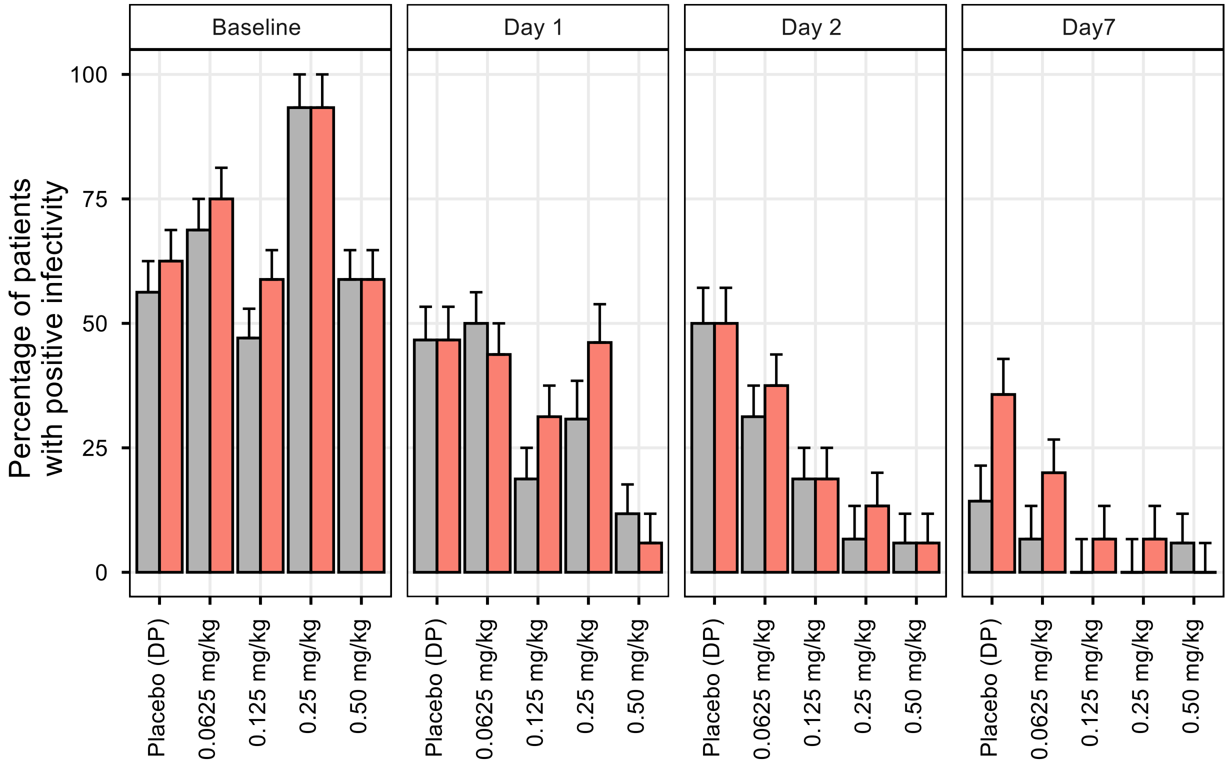

Figure S4. Observed (grey bars) and predicted percentage of patients with positive mosquito infectivity (light red bars) of each dosing arm regimen, stratified by day after primaquine administration.

Table S1 Baseline characteristics of participants, stratified by primaquine dose group

	**Overall (n=81)**	**Placebo (n=16)**	**0.0625 mg/kg (n=16)**	**0.125 mg/kg (n=17)**	**0.25 mg/kg (n=15)**	**0.5 mg/kg (n=17)**
Age (years)	12 (7-25)	22 (7-30)	10 (8-11)	22 (7-32)	10 (7-16)	18 (8-30)
Haemoblobin (g/L)	124 (112-138)	130 (110-139)	120 (108-129)	135 (111-144)	119 (112-132)	122 (113-142)
Gametocyte density by microscopy (/µL)	64 (32-720)	64 (32-144)	96 (48-576)	80 (32-320)	144 (48-720)	48 (32-192)
Gametocyte density by qRT-PCR (/µL)	123 (87-223)	119 (88-148)	137 (84-244)	95 (63-185)	266 (109-370)	103 (78-190)
Asexual parasite prevalence by microscopy	54 (67%)	8 (50%)	11 (69%)	14 (82%)	8 (53%)	13 (77%)
Asexual parasite density by microscopy (/µL)[a]	376 (64-2128)	176 (40-3840)	1088 (144-2832)	216 (48-1264)	1656 (432-9808)	144 (32-1792)
Mosquito infectivity (%)	5.41 (0-23.9)	2.58 (0-10.7)	6.18 (1.21-32.0)	0.645 (0-19.1)	30.7 (8.52-56.1)	1.48 (0-11.3)
Symptomatic malaria[b]	6 (7%)	0	3 (19%)	1(6%)	0	2 (12%)

Data are median (IQR) or n (%).

[a] Median parasite density were calculated for parasite-positive individuals only.

[b] Temperature ≥ 37.5°C and parasitemic.

References:

(1) Wampfler, R. *et al.* Strategies for detection of Plasmodium species gametocytes. *PLoS ONE* **8**, e76316 (2013).

(2) Ouédraogo, A.L. *et al.* A protocol for membrane feeding assays to determine the infectiousness of *P. falciparum* naturally infected individuals to *Anopheles gambiae*. *MalariaWorld Journal* **4**, 1-4 (2013).

(3) Keizer, R.J., van Benten, M., Beijnen, J.H., Schellens, J.H. & Huitema, A.D. Pirana and PCluster: a modeling environment and cluster infrastructure for NONMEM. *Comput Methods Programs Biomed* **101**, 72-9 (2011).

(4) Lindbom, L., Ribbing, J. & Jonsson, E.N. Perl-speaks-NONMEM (PsN)--a Perl module for NONMEM related programming. *Comput Methods Programs Biomed* **75**, 85-94 (2004).

(5) Goncalves, B.P. *et al.* Age, Weight, and CYP2D6 Genotype Are Major Determinants of Primaquine Pharmacokinetics in African Children. *Antimicrob Agents Chemother* **61**, (2017).

(6) Collins, K.A. *et al.* A controlled human malaria infection model enabling evaluation of transmission-blocking interventions. *J Clin Invest* **128**, 1551-62 (2018).

(7) Pasay, C.J. *et al.* Piperaquine Monotherapy of Drug-Susceptible Plasmodium falciparum Infection Results in Rapid Clearance of Parasitemia but Is Followed by the Appearance of Gametocytemia. *J Infect Dis* **214**, 105-13 (2016).

(8) Bolscher, J.M. *et al.* A combination of new screening assays for prioritization of transmission-blocking antimalarials reveals distinct dynamics of marketed and experimental drugs. *J Antimicrob Chemother* **70**, 1357-66 (2015).

(9) Ahn, J.E., Karlsson, M.O., Dunne, A. & Ludden, T.M. Likelihood based approaches to handling data below the quantification limit using NONMEM VI. *J Pharmacokinet Pharmacodyn* **35**, 401-21 (2008).

(10) Savic, R.M. & Karlsson, M.O. Importance of shrinkage in empirical bayes estimates for diagnostics: problems and solutions. *AAPS J* **11**, 558-69 (2009).

(11) Dosne, A.G., Bergstrand, M., Harling, K. & Karlsson, M.O. Improving the estimation of parameter uncertainty distributions in nonlinear mixed effects models using sampling importance resampling. *J Pharmacokinet Pharmacodyn* **43**, 583-96 (2016).

(12) Dosne, A.G., Bergstrand, M. & Karlsson, M.O. An automated sampling importance resampling procedure for estimating parameter uncertainty. *J Pharmacokinet Pharmacodyn* **44**, 509-20 (2017).

(13) Bergstrand, M., Hooker, A.C., Wallin, J.E. & Karlsson, M.O. Prediction-corrected visual predictive checks for diagnosing nonlinear mixed-effects models. *AAPS J* **13**, 143-51 (2011).

(14) Schneider, P. *et al.* Quantification of Plasmodium falciparum gametocytes in differential stages of development by quantitative nucleic acid sequence-based amplification. *Mol Biochem Parasitol* **137**, 35-41 (2004).
